# Gestational age-based reference ranges for cervical length and preterm birth prediction in triplet pregnancies: an observational retrospective study

**DOI:** 10.1186/s12884-022-04997-1

**Published:** 2022-08-30

**Authors:** Carlota Rodo, María de la Calle, Anna Maroto, Nerea Maiz, Silvia Arévalo, Pablo Garcia-Manau, Manel Mendoza, José Luis Bartha, Elena Carreras

**Affiliations:** 1 Maternal-Fetal Medicine Research Group, Department of Obstetrics, Vall d’Hebron Institut de Recerca (VHIR), Vall d’Hebron Hospital Universitari, Vall d’Hebron Barcelona Hospital Campus, Passeig Vall d’Hebron, 119-129, 08035 Barcelona, Spain; 2 Maternal-Fetal Medicine, Department of Obstetrics and Gynecology, Hospital Universitario La Paz-Carlos III, Madrid, Spain; 3 Maternal-Fetal Medicine, Department of Obstetrics, Hospital Universitari Dr Josep Trueta, Girona, Spain

**Keywords:** Triplet pregnancy, Cervical length, Preterm delivery, Gestational age-based reference ranges, Pessary, Cerclage

## Abstract

**Objectives:**

To develop gestational age-based reference ranges for cervical length in triplet pregnancies. The secondary objective was to assess the performance of cervical length measured between 18 and 20 + 6 days for the prediction of preterm delivery before 28 and 32 weeks, respectively.

**Methods:**

Observational retrospective study of triplet pregnancies in three Spanish tertiary-care hospitals between 2001 and 2019. Cervical length measurements were consecutively obtained between 15 and 34 weeks of gestation. Pregnancies undergoing multifetal reduction or fetal surgery were excluded.

**Results:**

Two hundred and six triplet pregnancies were included in the final analysis. There was a quadratic decrease in cervical length with gestational age. The median and fifth centiles for cervical length at 20 weeks were 35 and 13 mm. In the prediction of preterm birth < 28 weeks, for a false positive rate of 5%, and 10%, the detection rates were 40.9%, and 40.9%, respectively, and the prediction of preterm birth < 32 weeks, 22.0% and 26.0%, respectively.

**Conclusions:**

In triplet pregnancies, cervical length decreases with gestational age. The performance of cervical length at 18–20 + 6 in screening for preterm birth before 28 and 32 weeks is poor.

## Contribution

- **What are the novel findings of this work?**

The study describes the gestational age-based reference ranges for cervical length in triplet pregnancies.

The performance of cervical length at second trimester scan in screening for preterm birth in triplet pregnancies is poor.

- **What are the clinical implications of this work?**

This is the first study to describe gestational age-bases reference ranges for cervical length in triplet pregnancies.

## Introduction

Although perinatal mortality of multifetal gestations has improved, perinatal morbidity increases from singletons to higher order number of fetuses; and the major cause of these complications is preterm delivery. Prevention of preterm birth and its related morbidity is a goal not only because of the implications it may have for both the mother and the newborn, but also to reduce costs of preterm birth care.

Transvaginal ultrasound for cervical length (CL) measurement provides valuable information regarding the risk of preterm birth for singletons and twin gestations [1, 2]. This procedure has been widely accepted, and nowadays is commonly used as a standard of care. Cervical length in triplets seems to have a more rapid decline than in twins [3], but standards are required to establish normal and aberrant cervical changes in multifetal gestations.

Studies in singletons and twins propose a cervical length cut-off of 25 mm at 20 weeks to predict preterm birth [4, 5]; however, the best cervical length cut-off in triplets has not been established yet.

The main purpose of the study was to develop gestational age-based reference ranges for cervical length in triplet pregnancies. The secondary objective was to assess the performance of cervical length measured between 18 and 20 + 6 days for the prediction of preterm delivery before 28 and 32 weeks, respectively.

## Methods

This was an observational study of an unselected cohort of triplet pregnancies followed up in three tertiary-care centres in Spain: Hospital Universitari Vall d’Hebron in Barcelona, Hospital Universitario La Paz in Madrid, and Hospital Universitari Doctor Josep Trueta in Girona.

The study population were pregnant women attending one of the three hospitals for pregnancy care. Inclusion criteria were pregnancies with three live fetuses at the 11–13 week scan, regardless of the chorionicity. Exclusion criteria were pregnancies with less than three live foetuses at the 20-week scan, pregnancies undergoing fetal surgery, termination of pregnancy or miscarriage of the whole pregnancy, and when no cervical length measurements were available.

### Clinical protocol

Transvaginal measurement of cervical length was offered to all pregnant women carrying triplets, every two weeks, from 15 weeks’ gestation until delivery. Delivery was planned from 34 to 36 weeks’ gestation. Ultrasound examinations were performed by experienced obstetricians following the guidelines for cervical length measurement of the Fetal Medicine Foundation [6].

We studied 313 sets of triplets with data available from the first trimester onwards. Patient characteristics, medical and obstetrical history were recorded in their medical files. Both chorionicity and gestational age were determined at the first-trimester ultrasound, the latter from the measurement of the longest crown-rump length. Cervical pessary or cerclage were offered in cases of cervical length below 25 mm, in accordance with the local protocols for singletons and twins, without well-established criteria for triplets. Both were removed at delivery or before in the case of any side effects.

The study was approved by the Ethics Committee of Vall d’Hebron University Hospital (IRB number PR(AMI)416–2020, date of approval July 24^th^, 2020). No written informed consent was obtained as it was a retrospective study with de-identified data. There was no patient or public involvement. No funding was provided for the study.

### Statistical analysis

For the descriptive analysis, data are presented as absolute and relative frequencies for categorical variables, and as median and range for continuous variables. We calculated median, 5^th^ centile and 95^th^ centile for each week of gestation between 15 and 34 weeks from the raw data.

Analysis of repeated measures with a multilevel mixed-effects linear model (fixed effects and random effects) was performed. The fixed-effect component included up to second-order polynomial terms of gestational age, chorionicity and maternal factors. The random effect component included the intercept as well as linear effects of gestational age. We obtained age-based reference ranges for the cervical length according to gestational age for the whole population cohort. Quantile regression was performed to estimate the 5^th^ and the 95^th^ percentiles at specific gestational age points.

Logistic regression analysis was carried out to predict preterm delivery before 28 and 32 weeks with cervical length measurements between 18 and 20 + 6 days. For those women who had more than one measurement in this period, we chose the shortest measurement for the analysis. Predicted probabilities from logistic regression analyses were used to construct receiver–operating characteristic (ROC). The area under the ROC curve (AUC), best threshold (Youden’s method) detection rate (DR), false positive rate (FPR), positive predictive value (PPV) and negative predictive value (NPV) of screening by cervical length were estimated for preterm birth before 28 and 32 weeks, respectively.

The software R was used for the statistical analysis. The packages ‘lme4’, ‘quantreg’, and ‘pROC’ were used.

## Results

Between 2001 and 2019, 313 triplet pregnancies were identified, and 107 were excluded from the analysis: 70 had multifetal reductions before 20 weeks (58 iatrogenic and 12 spontaneous), 17 cases of twin-twin transfusion syndrome requiring fetal surgery, five cases of termination of pregnancy and 15 women with no cervical length data available. Two hundred and six pregnancies were included in the final analysis: 115 (55.8%) women from Barcelona, 75 (36.4%) from Madrid and 16 (7.8%) from Girona. One hundred and six pregnancies (51.5%) were trichorionic triamniotic pregnancies, 66 (32.0%) dichorionic triamniotic, 32 (15.5%) monochorionic triamniotic and two (1.0%) monochorionic diamniotic.

### Maternal demographics

Maternal demographic data could only be retrieved from 2010 onwards. The mean maternal age was 33.7 years (SD, 4.66), the median maternal weight was 64 kg (range, 40 to 103 kg), the median height was 164 cm (range, 150 to 185). 121 of 149 (81.2%) women were of Caucasian origin, two (1.3%) were Black, 13 (8.7%) North African, ten (6.7%) were Latin American, and three (2.0%) had other origins. Two out of 148 (1.4%) women had a previous preterm delivery. Seventy-three out of 189 (38.6%) pregnancies were conceived spontaneously and 116 (61.4%) by assisted reproduction techniques.

### Pregnancy outcomes

Pregnancy outcome was not available for four women and they were excluded for this part of the analysis. Two-hundred and two women were included in the analysis for preterm birth. The median gestational age at delivery was 33.0 weeks (IQR 31.1;34.1). Twenty-two women (10.9%) delivered before 28 weeks and 62 (30.7%) delivered before 32 weeks. A cervical pessary was placed due to cervical length less than 25 mm to 54 women (26.2%). The median gestational age at delivery in this group was 32.9 weeks (IQR 29.9;34.5). A cervical cerclage was placed to six women (2.9%). The median gestational age at delivery in this group was 27.1 weeks (IQR 25.0;30.1).

### Cervical length reference ranges

**Fig. 1:**
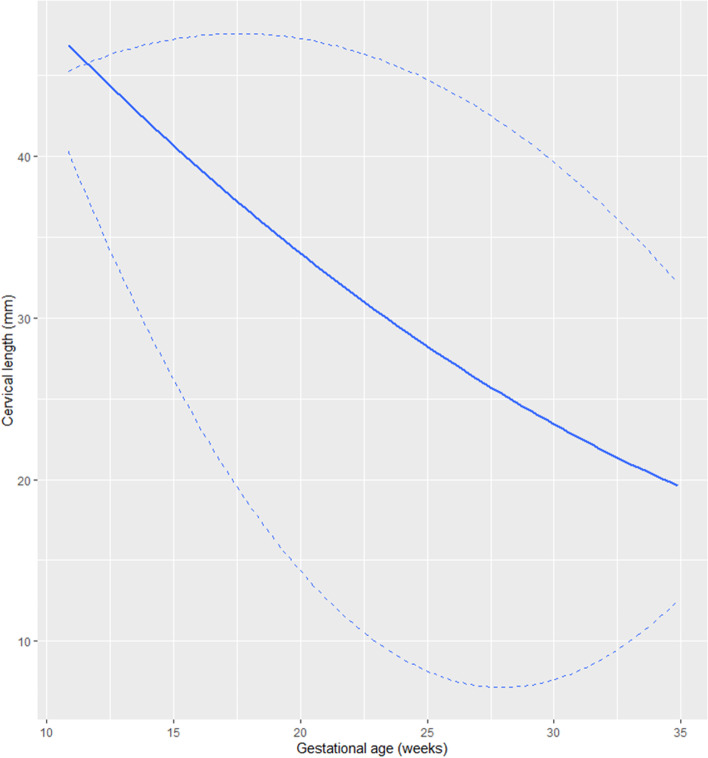
Gestational age-based reference ranges for cervical length

One-thousand one-hundred and sixty measurements of cervical length were obtained. There was a quadratic decrease in cervical length with gestational age. The median and fifth centiles for cervical length at 20 weeks were 34 and 13 mm. The median and fifth centiles at 28 weeks were 26 and 4 mm. And the median and fifth centiles at 32 weeks were 22 and 9.6 mm. The gestational age-based reference ranges for cervical length are shown in Fig. 1 and Table 1.

**Table 1:** Gestational age-based reference rages for cervical length in triplet pregnancies

Gestational age (weeks)	n	Observed mean	Observed SD	Observed 5th percentile	Observed Median	Observed 95th percentile	Estimated Median	Estimated 5^th^ percentile	Estimated 95^th^ percentile
15	19	39,7	5,5	30,5	41,0	45,3	40,2	24,8	47,3
16	18	38,1	5,2	30,9	37,0	46,3	38,8	22,1	47,5
17	22	36,0	5,9	25,3	35,5	44,9	37,4	19,7	47,6
18	32	37,8	6,9	29,0	38,0	47,0	36,0	17,5	47,6
19	52	37,3	6,8	28,6	38,0	48,0	34,7	15,5	47,5
20	77	34,1	9,0	13,0	35,0	45,0	33,5	13,7	47,3
21	61	34,1	10,3	13,0	36,0	47,0	32,2	12,1	47,0
22	60	31,9	13,2	6,7	34,5	47,1	31,1	10,8	46,5
23	71	30,0	10,9	13,0	33,0	45,0	29,9	9,6	46,0
24	95	29,1	12,0	6,7	31,0	45,3	28,8	8,7	45,4
25	73	23,6	10,7	9,2	22,0	40,0	27,8	8,0	44,6
26	86	26,3	12,4	5,0	28,0	45,8	26,8	7,5	43,8
27	91	25,1	10,7	9,0	26,0	44,0	25,8	7,2	42,8
28	74	24,3	11,2	4,0	25,5	41,0	24,9	7,1	41,8
29	95	24,6	10,0	9,7	25,0	39,0	24,0	7,3	40,6
30	63	22,8	9,2	8,2	22,0	37,9	23,1	7,6	39,4
31	85	22,4	9,6	10,0	22,0	36,8	22,3	8,2	38,0
32	52	21,9	8,9	9,6	22,0	37,5	21,6	9,0	36,6
33	33	22,9	8,2	10,6	25,0	35,0	20,8	10,0	35,0
34	3	23,7	8,5	15,3	27,0	29,7	20,1	11,2	33,3

*n* Number of measurements acquired

The cervical length throughout gestation was not affected by chorionicity (*p* = 0.943), ethnic origin (*p* = 0.468), maternal age (*p* = 0.297), weight (*p* = 0.663), height (*p* = 0.468), mode of conception (*p* = 0.991), or previous preterm delivery (*p* = 0.056).

### Cervical length dynamics

**Fig. 2:**
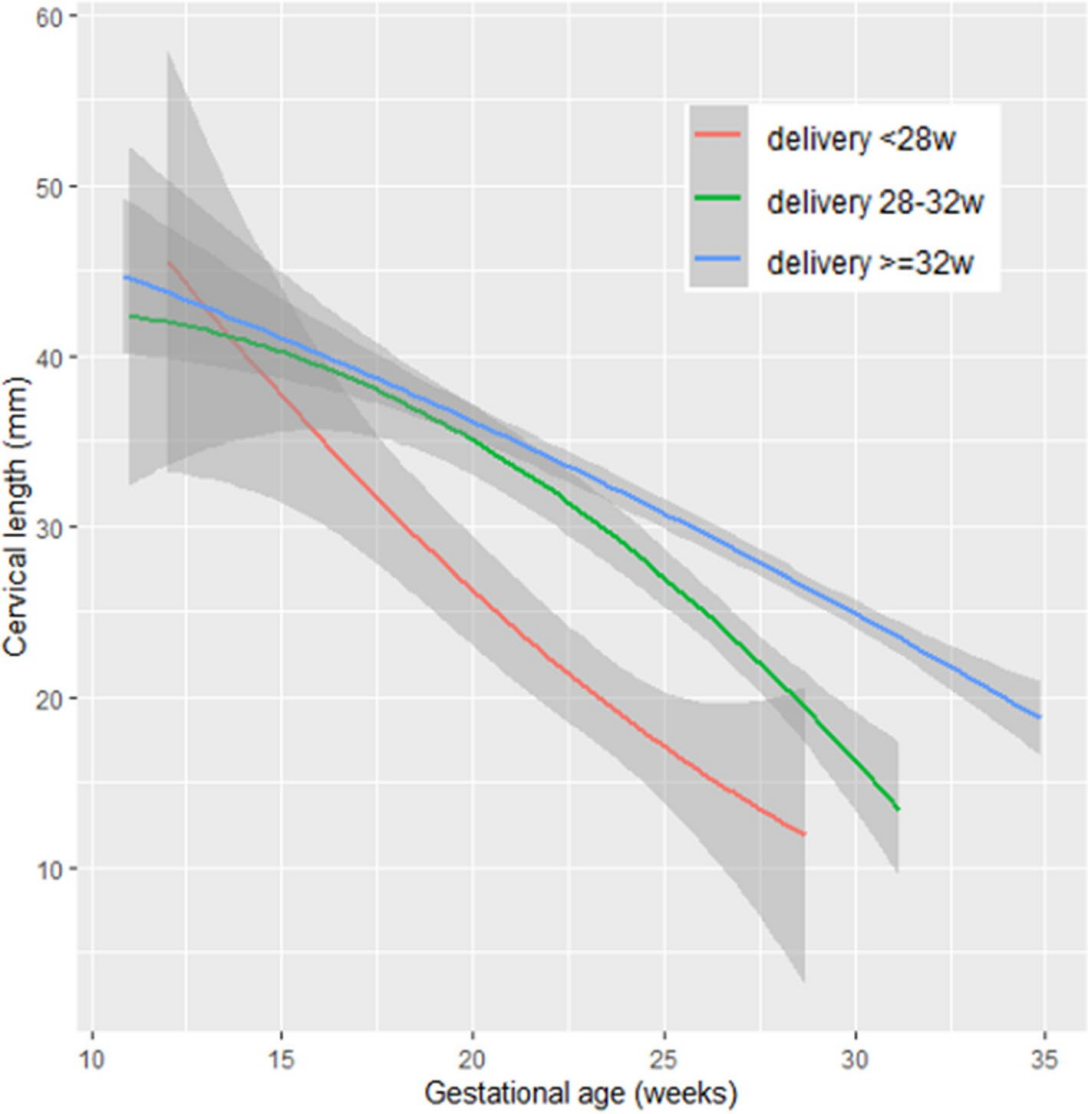
Cervical length dynamics for preterm delivery before 28 weeks’ gestation, between 28 and 32 weeks, and after 32 weeks’ gestation

Figure 2 shows that below 17 weeks there were no differences in CL for women delivering before 28 weeks, between 28 and 32 weeks or after 32 weeks. For women delivering before 28 weeks, cervical length slope is steeper from 17 weeks onwards (red line). For women delivering between 28 and 32 weeks or after 32 weeks, there were no differences in CL until 20 weeks. From 20 weeks onwards, the slope is steeper for women delivering between 28 and 32 weeks and is significantly shorter after 24 weeks (green line).

### Preterm birth prediction

**Fig. 3:**
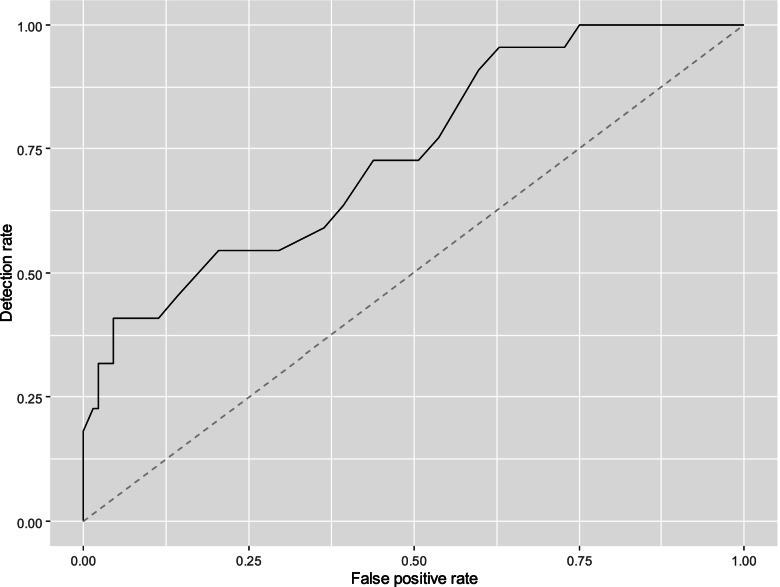
ROC curve for prediction of preterm delivery at 28.0 weeks’ gestation

**Fig. 4:**
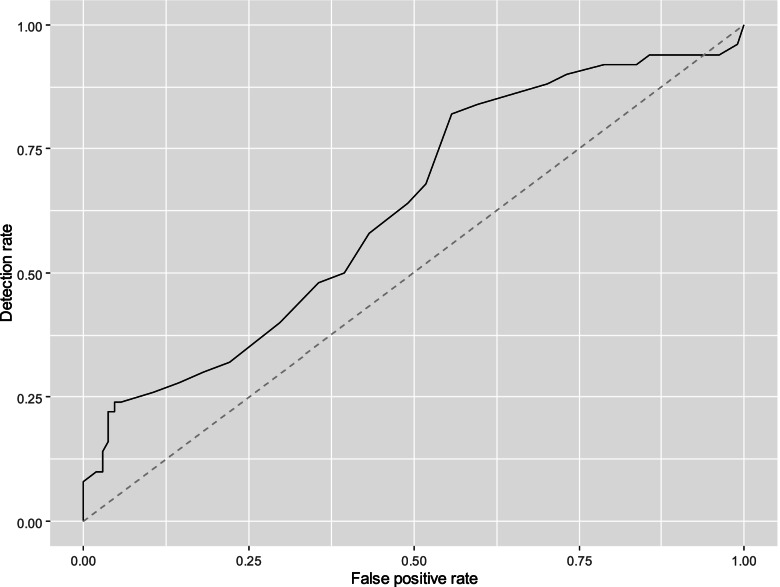
ROC curve for prediction of preterm delivery at 32.0 weeks’ gestation

There were 154 women with cervical length measurements available between 18 and 20 + 6 weeks of gestation. The AUC for the prediction of preterm birth before 28 and 32 weeks were 0.739 (95% CI, 0.626–0.852), and 0.629 (95% CI, 0.535–0.724), respectively. ROC curves are shown in Figs. 3 and 4. The DR, PPV and NPV for a fixed FPR of 5%, and 10% and for the best threshold are shown in Table 2. For the prediction of preterm birth before 28 weeks, the cervical length threshold for a 5% FPR is between 24.5 and 25.5 mm, and between 27.5 and 28.5 mm for a FPR of 10%. For the prediction of preterm birth before 32 weeks, the cervical length threshold for a 5% FPR is between 26.5 and 27.5 mm, and between 27.5 and 28.5 mm for a FPR of 10%.

**Table 2:** Performance of cervical length in screening for preterm birth before 28 and 32 weeks

	Prediction of delivery before 28 weeks			
Threshold	FPR	DR (95% CI)	PPV (95% CI)	NPV (95% CI)
NA	5%	40.9% (18.2–59.1%)	54.8% (31.3–66.3%)	90.0% (86.8–93.3%)
NA	10%	40.9% (22.7–63.6%)	40.5% (27.5–51.5%)	90.1% (87.5–93.7%)
24.5	4.5% (1.5–8.3%)	40.9% (22.7–63.6%)	61.1% (38.5–84.6%)	90.7% (88.0–93.9%)
	Prediction of delivery before 32 weeks			
NA	5%	22.0% (7.2–36.0%)	67.9% (40.9–77.6%)	71.7% (68.0–75.5%)
NA	10%	26.0% (14.4–38.8%)	55.6% (40.9–65.1%)	71.7% (68.6–75.4%)
38.5	55.8% (46.2–66.4%)	82.0% (70.0–92.0%)	41.4% (36.046.9%)	83.9% (74.5–92.1%)

*FPR* False positive rate, *DR* Detection rate, *PPV* Positive predictive value, *NPV* Negative predictive value, *CI* Confidence interval, *NA* Not available

## Discussion

### Main findings

This study describes the gestational age-based reference ranges for cervical length in triplet pregnancies in a Spanish population from three different settings. Cervical length decreased with gestational age, but was not affected by chorionicity, mode of conception or previous preterm delivery. The performance of cervical length measured between 18 and 20 + 6 weeks for prediction of preterm birth before 28 and 32 weeks is poor.

### Interpretation

Our group previously published the gestational age-based reference ranges for cervical length for singletons and twins in the Spanish population [4]: the mean cervical length at 20 weeks for singletons and twins is 38 and 36 mm, respectively, which is similar to 35 mm for triplets. However, the largest differences are observed for the 5^th^ centile, being 26 mm and 24 mm for singletons and twins, respectively, and 13 mm for triplets.

Others demonstrated that the shorter the cervical length, the higher the risk of preterm delivery, and the same applies to triplets [7]. According to other publications, the median cervical length before 24 weeks in triplet pregnancies is 34–35 mm [7, 8], which is similar to our results.

Cervical length is a poor test for predicting preterm delivery in triplets [8]. The cervical length cut-offs at 18–20 + 6 weeks that best predicted preterm delivery at 28.0 and 32.0 weeks were 24 mm and 38 mm, respectively. However, sensitivity and specificity for both measurements were low. Fichera et al. [8] analysed the performance of cervical length at mid trimester to predict preterm delivery at 28 and 32 weeks. Our results are consistent with theirs. In the prediction of preterm birth before 28 weeks in our cohort for a FPP of 10% the detection rate was 40.9% and in Fichera’s study the detection rate was 41.7% for a FPP of 16.7%. Similarly, in the prediction of preterm birth before 32 weeks, for a FPP of 10%, our detection rate was 26% and in Fichera’s study, the detection rate was 29.3% for a FPP of 14%. This suggests that the risk of premature delivery is subjected to stressors other than cervical shortening, also in triplet pregnancies.

Concerns arise regarding the use of cervical length measurement in triplets due to its limited predictive value to predict preterm delivery [9] and the lack of effective interventions to prevent preterm delivery [10]. There is little data published on the use of pessary or cerclage in triplets and, for a 38 mm or 24 mm cut-off respectively, no benefit was found in prolonging pregnancy [11, 12]. Other strategies to prevent preterm delivery in multiples, such as progesterone or bed rest, have also proven to be ineffective [13, 14].

### Strengths and limitations

The major strength of this study is the large number of cervical length measurements obtained to perform the gestational age-based reference ranges. There are some limitations: First, women with shorter cervical length were followed closely; therefore, more cervical length measurements were performed in these women, which might have biased the curve towards lower values. Second, we could only retrieve demographic data from 2010 onwards; however, we do not believe it might have impacted the results.

We decided not to exclude women carrying cerclage or pessary because this is an observational study and neither of these techniques have shown any benefits in prolonging pregnancy [11, 12]; however, the placement of a cerclage or pessary could also have altered the trend of the curve.

In triplet pregnancies, cervical length decreases with gestational age. The fifth centile for cervical length at 18–20 + 6 weeks is 13 mm. The performance of cervical length at 18–20 + 6 in the screening for preterm birth before 28 and 32 weeks is poor.

## Data Availability

The datasets used during the current study are available from the corresponding author on reasonable request.

## Abbreviations

Kg: Kilogram
Cm: Centimeters
Mm: Milimeters
CI: Confidence interval
ROC: Receiver–operating characteristics
DR: Detection rate
PPV: Positive predictive value
NPV: Negative predictive value
FPR: False-positive rate
